# Terpenic fraction of *Pterodon pubescens* inhibits nuclear factor kappa B and extracellular signal-regulated protein Kinase 1/2 activation and deregulates gene expression in leukemia cells

**DOI:** 10.1186/1472-6882-12-231

**Published:** 2012-11-27

**Authors:** Monica Farah Pereira, Thiago Martino, Sergio Ranto Dalmau, Márcia Cristina Paes, Christina Barja-Fidalgo, Rodolpho Mattos Albano, Marsen Garcia Pinto Coelho, Kátia Costa de Carvalho Sabino

**Affiliations:** 1 Departamento de Bioquímica, Instituto de Biologia Roberto Alcantara Gomes, Universidade do Estado do Rio de Janeiro, Av. 28 de Setembro 87, fds, PAPC, Vila Isabel, Rio de Janeiro, RJ, CEP 20551-030, Brazil; 2 Departamento de Farmacologia, Instituto de Biologia Roberto Alcantara Gomes, Universidade do Estado do Rio de Janeiro, Rio de Janeiro, Brazil

**Keywords:** *Pterodon pubescens* seeds, Leukemia, Cell cycle, Cell signaling

## Abstract

**Background:**

Plant derived compounds have been shown to be important sources of several anti-cancer agents. As cell cycle deregulation and tumor growth are intimately linked, the discovery of new substances targeting events in this biochemical pathway would be of great value. The anti-leukemic effect of an ethanolic extract of *Pterodon pubescens* seeds (EEPp) has been previously demonstrated and now we show that a terpenic subfraction (SF5) of EEPp containing farnesol, geranylgeraniol and vouacapan derivatives induces apoptosis in the human chronic myelogenous leukemia cell line K562. This work addresses SF5’s antiproliferative mechanisms in these cells since they are still unclear.

**Methods:**

DNA synthesis in K562 cells was assessed by [^3^H]-methyl-thymidine incorporation and cell cycle status by flow cytometry. The expression of cyclins D1 and E2, of the cell cycle inhibitor p21 and of the proto-oncogene c-myc was evaluated by semi-quantitative RT-PCR. Extracellular-signal-regulated kinases (ERK) 1/2 and nuclear factor kappa B (NF-κB) activation was evaluated by western blotting.

**Results:**

In K562 cells, SF5 treatment induced a higher inhibition of DNA synthesis and cell growth than the original EEPp hexanic fraction from which SF5 originated, and also arrested the cell cycle in G1. Exposure of these cells to SF5 led to a decrease in cyclin E2 and c-myc expression while p21 mRNA levels were increased. Furthermore, SF5 inhibited the activation of mitogen-activated protein kinase (MAPK) ERK 1/2 and NF-κB.

**Conclusions:**

This work suggests that the anti-leukemic action of SF5 is linked to the inhibition of ERKs, NF-κB and c-myc signaling pathways resulting in reduced cyclin E2 mRNA expression and cell cycle arrest in the G1 phase.

## Background

Cancer is one of the leading causes of death for both men and women. Therefore, the search for new anti-tumor agents has been increasing in recent years. As cell cycle deregulation and tumor growth are intimately linked [1,2], the discovery of new substances targeting events in this biochemical pathway would be of great value. In this light, the search for natural compounds of plant origin combined to pharmacological investigations could reveal new substances with potential anti-cancer properties.

Eukaryotic cell cycle progression is tightly regulated by the sequential activation and inactivation of cyclin-dependent kinases (CDKs), being positively and negatively regulated by cyclins and cyclin-dependent kinase inhibitors, respectively [2,3]. Cyclins D and E are fundamental G1-phase cyclins, while cyclins A and B allow cells to traverse the S and G_2_M phases of the cell cycle, respectively. Inappropriate expression, regulation and/or mutations of CDKs, cyclins and their regulatory proteins have been described in various cancer types [1].

An important aspect of the cell cycle is that it is controlled by intricate intracellular signaling mechanisms. One of them is the MAPK pathway, which is formed by a cascade of kinases, generally referred to as Raf/MEK/ERK. In this pathway, activated extracellular signal-regulated kinases (ERKs) translocate into the nucleus and regulate the activity of transcription factors such as c-myc and others [4,5], resulting in the expression of genes required for cell cycle progression like cyclins D1 and E and p27 [6,7]. In addition, the expression of these cyclins and of other cell cycle related genes is also stimulated by the NF-κB transcription factor [8,9] which also influences the epigenetic regulation of gene transcription by increasing, for example, the expression of O^6^-methylguanine DNA methyltransferase, an important DNA damage repair enzyme [10].

The anti-tumor effects of terpene derivatives have been demonstrated in medicinal plants [11]. *Pterodon pubescens* (Pp) Benth. (Leguminosae), popularly known as “Sucupira branca”, is a native tree widely distributed over the central region of Brazil, whose seeds are used in folk medicine to treat rheumatic and inflammatory diseases [12]. These properties have been scientifically confirmed and, recently, additional anti-tumor effects have also been demonstrated for *Pterodon pubescens* seeds [13-15]. Phytochemical studies with the hexanic fraction of the ethanolic extract of *Pterodon pubescens* seeds (EEPp) have shown the presence of linear terpenes such as farnesol and geranylgeraniol, and of diterpenes with a vouacapan skeleton [16-19]. The SF5 hexanic subfraction of EEPp is rich in farnesol and vouacapan and geranylgeraniol diterpene derivatives and recent work reported that SF5 shows anti-tumor properties by inducing apoptosis in leukemia cells [19]. As it is still unclear whether SF5 affects the cell cycle, this work was undertaken to investigate the effects of this terpenic subfraction on the proliferation of leukemia cells and its mechanism of action.

## Methods

### Materials and reagents

RPMI 1640 medium, streptomycin sulfate, propidium iodide, triton X-100, RNAse, sodium dodecyl sulfate, PPO, POPOP, Phytohemagglutinin A (PHA), protease inhibitors and serum bovine albumin were purchased from Sigma Chemical Co., USA. Penicillin was purchased from Fontoura-Wyerth, Brazil. Fetal calf serum (FCS) was purchased from Cultilab, Brazil. ^3^H-metil-thymidine was purchased from Amersham Life Science, USA. For PCR, dNTP mix, Trizol, first-strand buffer, ribonuclease inhibitor, DTT, SuperScriptTM II RNAse H Reverse Transcriptase, random primers, MgCl_2_ and Taq polymerase platinum were purchased from Invitrogen, USA. Primers for cyclins D1 and E2, c-myc, p21 and beta actin were purchased from Imprint, Brazil. Anti-human antibodies: p65, Histone, ERK 1/2 and p-ERK 1/2 were purchased from Santa Cruz Biotechnology, USA. ECL kit and film for western blotting were purchased from Amersham Biosciences, USA.

### Plant material collection and extraction

*Pterodon pubescens* seeds were collected by Luciana Pontes Coelho in Goiás, Brazil and flowering branches of this tree were used for taxonomic identification by Haroldo Cavalcante de Lima at the Departamento de Botânica Sistemática, Jardim Botânico do Estado do Rio de Janeiro, Rio de Janeiro, RJ, Brazil, where a voucher of the collected specimen has been deposited (RB 350279). *Pterodon pubescens* seeds were powdered in liquid nitrogen and submitted to 100% ethanol extraction (15 g/100 mL) at room temperature for 15 days as described by Silva and collaborators [12]. The ethanolic extract of *Pterodon pubescens* seeds (EEPp) was obtained (50% yield, w/w) after ethanol evaporation. Afterwards, the EEPp was fractionated by liquid-liquid partition chromatography using hexane. The obtained hexanic fraction (HEX, 55% yield) was submitted to a column chromatography (42 × 2.5 cm) on silica gel (Art. 7733 – Kieselgel 60, 35–70 mesh, 0.2-0.5 mm, Merck) and eluted successively with hexane (500 mL), hexane/dichloromethane (1:1) (500 mL), hexane/ethyl acetate (1:1) (500 mL), ethyl acetate (500 mL), ethanol (500 mL) and 2% acetic acid in ethanol (500 mL), as previously described by Pereira and collaborators [19]. After gas chromatography analysis of eluted samples, they were joined in eight subfractions according to their chromatographic profile similarity. The HEX GC profile shows two regions of important peaks, the first one in the retention time range of 2.0 and 5.0 seconds and the second one between 6.0 and 10.0 seconds. The GC analysis of the most cytotoxic subfraction to leukemia cells, subfraction 5 (SF5, 32.9% of HEX fraction), indicates that it corresponds to the second region of the HEX fraction, containing the furane diterpenes methyl-6α-acetoxy-7β-hydroxy-vouacapan-17β-oate and 6α,7β-diacetoxy-vouacapan- besides epoxyfarnesol and geranylgeraniol derivatives [20] HEX and SF5 were previously diluted at different concentrations with RPMI 1640 supplemented with 10% FCS.

### Cell proliferation assays

The human chronic myelogenous leukemia cell line K562 (CCL-243) was purchased from the American Type Culture Collection (ATCC) and cultured in RPMI 1640 medium supplemented with 10% fetal calf serum. The effects of the plant derived samples on cell growth were evaluated by treatment of cells (2.5 × 10^5^ cells/mL) with HEX or SF5 at different concentrations for up to 72 h at 37°C and 5% CO_2_. Viable cells were counted in a 12 h interval using the trypan blue dye exclusion method. To assess DNA synthesis, 25 μL/well of 10 μCi/mL [^3^H]-methyl-thymidine (^3^H-Tdr; Amersham Biosciences, Brazil) were added for the last 24 h of culture. Then, the cells were harvested on filter paper and processed for the determination of of ^3^H-Tdr incorporation by liquid scintillation.

### Cell cycle analysis

To determine the distribution of DNA content, K562 cells were treated with DNA staining solution as previously described [19]. Cells (2.5 × 10^5^/mL) were incubated either in the absence (control) or in the presence of HEX or SF5 at 30 μg/mL for 36 h at 37°C and 5% CO_2_. After centrifugation (400 x g) for 5 min, 1 × 10^6^ viable cells were suspended in DNA staining solution (0.3% Triton X-100 and 50 μg/mL propidium iodide [PI] in 43 mM citrate buffer) and maintained for 15 min at room temperature in the dark. Later, the samples were treated with 50 μg/mL ribonuclease A (Sigma Chemical Co., St. Louis, MO, USA) in 43 mM citrate buffer pH 8.2 for 15 min at room temperature. PI fluorescence was measured (100,000 events per sample) using a FACSCalibur flow cytometer (Becton Dickson, USA). The excitation wavelength for PI was 488 nm and the emission detected at 585 ± 15 nm. Analysis was done with the WinMDI 2.8 software.

### Imunoblotting

Cells (2.5 × 10^5^ cells/mL) were incubated with 50 μg/mL SF5 for different times. The whole cell lysate was prepared for the evaluation of phosphorylated (p-ERK 1/2) and total ERK 1/2 protein expression. Cells were washed twice in PBS, suspended in lysis buffer (50 mM HEPES pH 6.4, 1 mM MgCl_2_, 10 mM EDTA, and 1% Triton X-100), left 30 min on ice and centrifuged at 4°C for 10 min. The supernatant was stored in a freezer. Nuclear extracts were prepared as previously described [20] for the evaluation of NF-κB activation. Protein concentration was measured by the Lowry assay [21] using bovine serum albumin as standard. Proteins were denatured for 5 min in sample buffer (glycerol, β-mercaptoethanol, 10% SDS, 10 N NaOH and bromophenol blue) and 30 μg of protein per sample were resolved in a 12% or 15% sodium dodecyl sulphate (SDS)-polyacrylamide gel electrophoresis (PAGE) and transferred to a nitrocellulose membrane (Bio-Rad). Blots of nuclear (for NF-κB and histone) or whole cell extracts (for p-ERK 1/2 and total ERK 1/2) were blocked for 2 h at room temperature with 2% BSA and 0.1% Tween in PBS and then incubated overnight with the specific primary antibodies anti-p65 or anti-pERK 1/2 in PBS containing 0.5% BSA and 0.1% Tween. The membranes were washed three times, soaked for 1 h with the secondary antibody (horseradish peroxidase-linked anti-mouse IgG), and washed again with PBS-Tween 0.1%. Finally, blots were developed using the enhanced chemiluminescence method (ECL; Amersham). Blots were reincubated with anti-histone or anti-ERK 1/2 as loading control of nuclear or whole cell extracts, respectively.

### RT-PCR for mRNA expression analysis

Total RNA of samples was extracted with the Trizol reagent according to the manufacturer’s instructions (Invitrogen). RNA samples were treated with DNAse I and RNA integrity was confirmed by denaturing agarose gel electrophoresis. Reverse transcription reactions were performed as previously described [15]. cDNAs were used in 25 μL mixtures of PCR buffer, 0.2 mM of each dNTP, 1.5 mM MgCl_2_, 25 pmoles of each primer, and 1.0 U of Taq DNA polymerase platinum (Invitrogen). Primers, annealing temperature and cycles used were: β-actin forward primer: 5^′^-TCCTGTGGCATCCACGAAACT-3^′^, reverse primer: 5^′^-GAAGCATTTGCGGTGGACGAT-3^′^ (59°C, 30 cycles, 314 pb); Cyclin D1 forward primer: 5^′^-TTGCTGCCCTTCTCCATGAT-3^′^, reverse primer: 5^′^-TCCCAACTGAAACCCAATGC-3^′^ (55°C, 30 cycles, 334 pb); Cyclin E2 forward primer: 5^′^-GATTTGTCCTTGGAGAACGG-3^′^, reverse primer: 5^′^-TTGGGTGTTGGTTCTTTGGTT-3^′^ (57°C, 30 cycles, 499 pb), p21^CIP/KIP^ forward primer: 5^′^-TTGCTGCCCTTCTCCATGAT-3^′^, reverse primer: (55°C, 30 cycles, 334 pb); Cyclin E2 forward primer: 5^′^-TCCCAACTGAAACCCAATGC-3^′^ (55°C, 30 cycles, 334 pb), c-myc forward primer: 5^′^-GTCCTCGGATTCTCTGCTC-3^′^, reverse primer: 5^′^-GACTCTGACACTGTCCAACT-3^′^, (60°C, 30 cycles, 342 pb). All genes examined were normalized to the housekeeping gene β-actin. PCR was performed in a Perkin Elmer GeneAmp PCR System 9600. Each PCR cycle consisted of an initial denaturation step at 94°C for 5 min followed by the appropriate number of cycles consisting of 94°C for 30 s, annealing at specific temperature for 30 s, and extension at 72°C for 1 min. Semi-quantitative analysis of the products was performed by 2% agarose gel electrophoresis and band intensities were determined by Lab Image software (Germany).

### Statistical analysis

Significant differences between pairs of groups were accessed using Student’s *t* test, with a significance level set at p < 0.05.

## Results

### Effects of SF5 on leukemic cell growth

**Figure 1:**
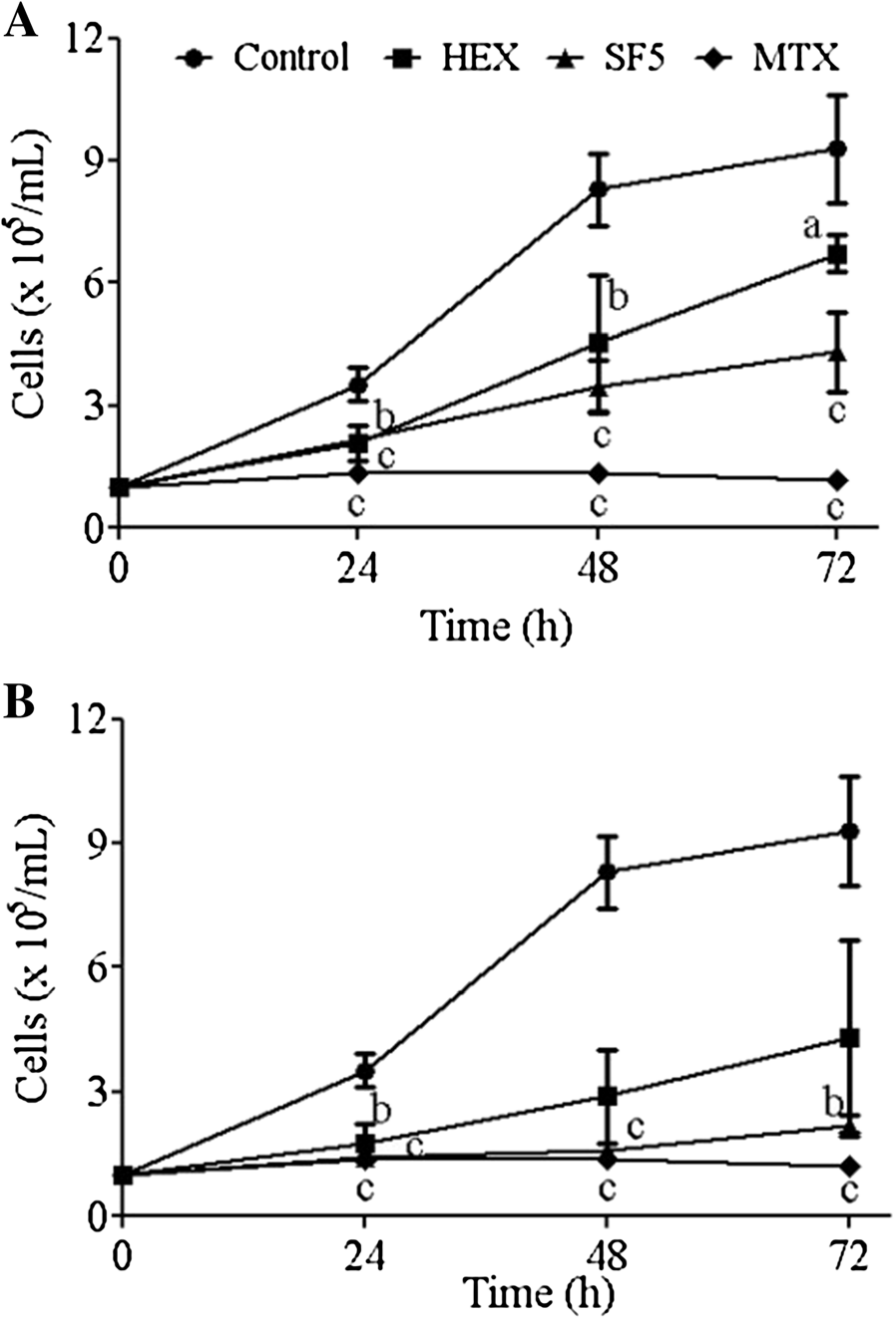
**Effects of hexanic fraction (HEX) and SF5 hexanic subfraction on K562 leukemia cell growth.** (**A**) 30 μg/mL, (**B**) 50 μg/mL. Cells (2.5 × 10^5^/mL) were cultured for 24, 48 and 72 h either in the absence (control) or presence of HEX, SF5 or methotrexate (MTX, 50 μg/mL). Viable cells were counted by Trypan blue dye exclusion. Results represent the mean ± SD of three independent experiments. ^a^p < 0.05, ^b^p < 0.01 and ^c^p < 0.001, versus control by Student’s *t* test.

HEX subfractionation of EEPp resulted in a subfraction (SF5) with greater inhibitory effect on cell growth. SF5 treatment reduced K562 cell growth in a time and concentration  dependent  manner.  As  shown  in  Figure 1,     signi-ficant reductions of 51.9 ± 12.7%, 66.2 ± 7.8% and 57.7 ± 19.6% in cell growth were observed after treating cells with 30 μg/mL SF5 for 24 h, 48 h and 72 h, respectively, compared to control cultures. This inhibition increased to 83.6 ± 4.1%, 91.9 ± 1.5% and 85.4 ± 3.9%, respectively, with SF5 at 50 μg/mL. The significant inhibition indexes of SF5 at 24 h and 48 h were similar (p > 0.05) to those of the traditional antineoplasic agent MTX (84.8 ± 7.0%, 95.2 ± 1.8% and 97.7 ± 24.5% at 24 h, 48 h and 72 h, respectively).

### Effects of SF5 on DNA synthesis and on the cell cycle

**Figure 2:**
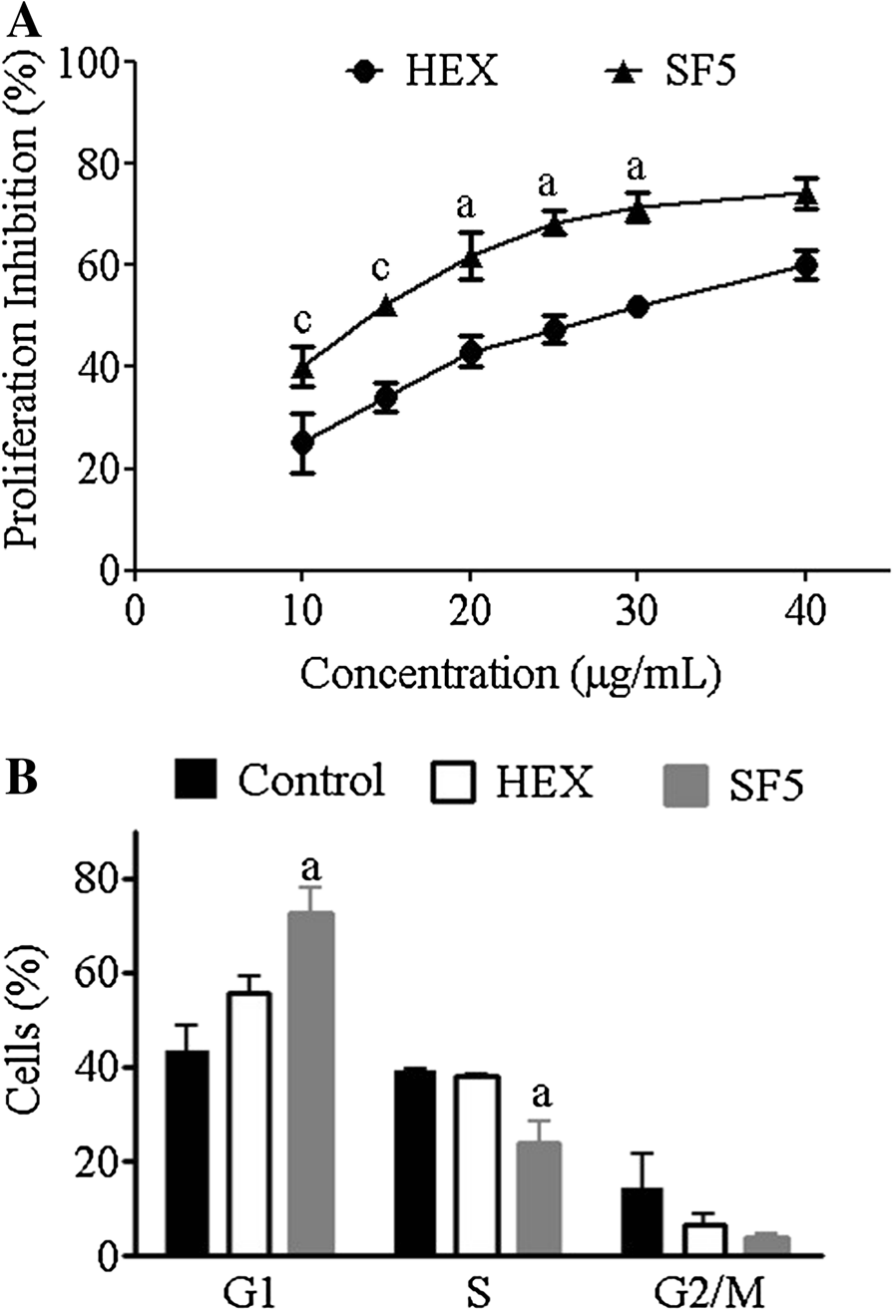
**Effects of hexanic fraction (HEX) and SF5 hexanic subfraction on K562 cell proliferation.** (**A**) ^3^H-methyl-thymidine incorporation into DNA. (**B**) Cell cycle analysis. Cells (2.5 × 10^5^/mL) were cultured for 72 h in the absence (control) or presence of different HEX or SF5 concentrations. To each well, 0.25 μCi of ^3^H-methyl-thymidine was added 24 h before the end of cultures. For cell cycle analysis cells were treated with HEX or SF5, both at 30 μg/mL, for 36 h. Hypodiploid nuclei and debris were discarded from the analysis. Results express means ± SD of three independent experiments. All inhibition indexes were significantly different from control cultures. ^a^p < 0.05 and ^c^p < 0.001 versus HEX fraction by Student’s *t* test.

Since SF5 treatment inhibited the growth of K562 leukemic cells, its effect on DNA synthesis (Figure 2A) and on the cell cycle (Figure 2B) was analyzed. Both HEX and SF5 significantly inhibited DNA incorporation of ^3^H-methyl-thymidine in K562 cells when compared to control cultures with SF5 showing higher inhibitory indexes on cell proliferation than the original HEX fraction (Figure 2A). The cell cycle profile of K562 leukemic cells was modified by treatment with SF5 (30 μg/mL). Cells accumulated in the G1 phase with concomitant reduction in the S and G2/M phases (Figure 2B). Again, SF5 induced a more intensive anti-leukemic effect than the original HEX fraction. However, the mechanisms underlying this effect remain unknown.

### Effects of SF5 on gene expression

**Figure 3:**
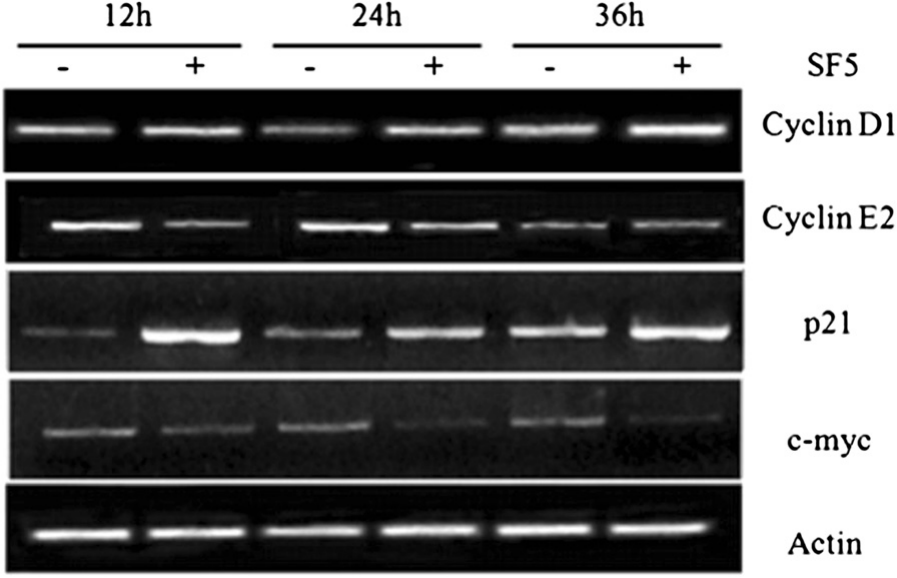
**Effects of SF5 on the expression of cyclins, p21 and c-myc in K562 cells.** Cells (2.5 × 10^5^/mL) were cultured in the absence (control) or presence of SF5 (50 μg/mL) for 36 h. RNA was extracted and the RT-PCR products were visualized by agarose gel electrophoresis as described in “Methods”. β-Actin mRNA was used as loading control.

Cyclins play a key role in controlling cell cycle progression by positively regulating CDKs activities at appropriate time points of the cell cycle. Since D1 and E2 cyclins regulate the transition from G1 to the S phase and SF5 induced G1 arrest in K562 cells (Figure 2B), these expression of these cyclins was analyzed after treating these cells with SF5 (50 μg/mL). Treatment with this subfraction increased cyclin D1 mRNA levels at all times studied while it reduced the expression of cyclin E2 after 12 and 24 h (Figure 3) in K562 cells. Cyclin-dependent kinase inhibitors (CKIs) control cell cycle progression by negatively regulating CDKs activities. In this work, SF5 treatment markedly increased p21 mRNA levels in K562 cells (Figure 3). Concomitantly, this treatment also led to a reduction in the expression of the cell survival and proliferation up-regulator transcription factor c-myc (Figure 3).

### Effects of SF5 on ERK 1/2 and NF-κB activation

**Figure 4:**
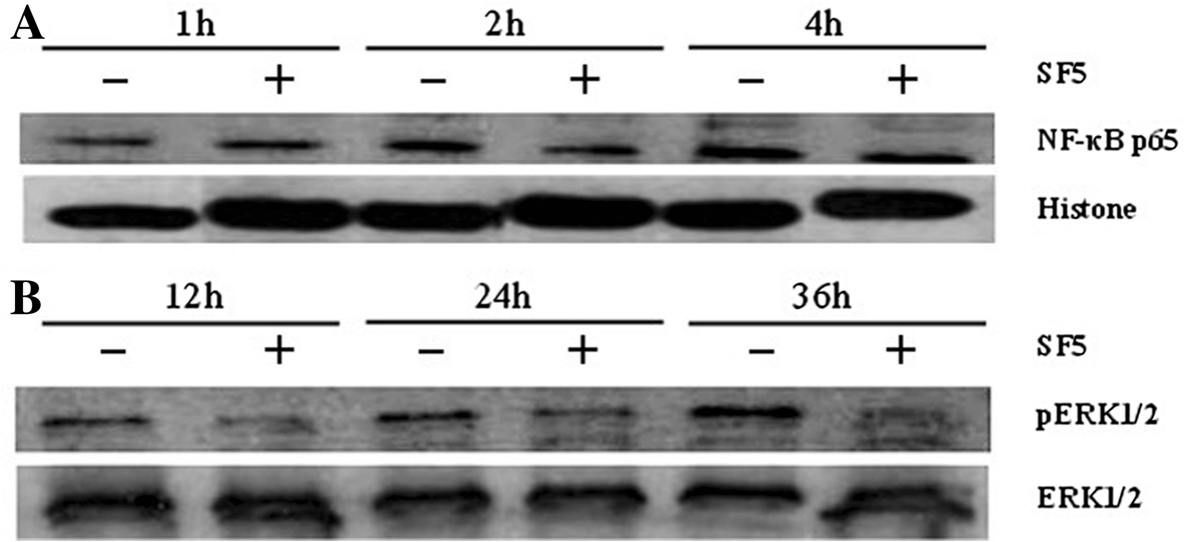
**Effects of SF5 on the activation of NF-κB and ERK 1/2.** (**A**) NF-κB protein nuclear translocation. (**B**) Phosphorylated ERK expression. Nuclear extracts (NF-κB) and whole cell lysates (pERK) were prepared as described in “Material and Methods”, from K562 cells (2.5 × 10^5^/mL) treated with SF5 (50 μg/mL) for the indicated times. Proteins (50 μg) were resolved by SDS-PAGE, transferred to nitrocellulose membranes, probed with anti-p65 or anti-p-ERK and developed by chemiluminescence. Histone was used as a loading control for nuclear extracts and total ERK 1/2 for whole cell lysates.

The effects of SF5 treatment on cell proliferation related intracellular signaling were evaluated by determining the levels of phosphorylated-ERK 1/2 and the nuclear translocation of the transcription factor NF-κB. Control cultures showed a lower level of nuclear NF-κB expression at 1 h, which was progressively increased at 2 and 4 h while K562 cells treated with SF5 at 50 μg/mL showed reduced nuclear NF-κB expression at 2 h and 4 h (Figure 4A). The level of total ERK 1/2 protein expression is higher than the phosphorylated form in all culture conditions (Figure 4B) and treatment with SF5 inhibited led to an inhibition of ERK 1/2 phosphorylation (Figure 4B).

## Discussion

Previous studies with the EEPp reported an anti-leukemic effect by arresting K562 cell cycle in the G1 phase [15]. Similar results were also demonstrated for melanoma cells [18]. SF5, a terpenic subfraction obtained after extracting EEPp with hexane, contains epoxyfarnesol (32%), geranylgeraniol (4.6%) and the 7β-acetoxy-vouacapan (1.9%) derivatives, methyl 6α-acetoxy-7β-hydroxy-vouacapan-17β-oate (6%) and 6α-7β-diacetoxy-vouacapan-14β-oate (20.6%) [19]. Previous studies reported cytotoxic effects and apoptosis induction in leukemia cells by treatment with SF5 [19]. Now this work demonstrates that SF5 treatment inhibits leukemia cell proliferation by down-regulating cell growth stimulating transcription factors and ERK 1/2 activation and by altering the expression of cell cycle regulators. Comparative analysis of the anti-proliferative effects of SF5 with the hexanic fraction of EEPp (HEX) from which it was obtained showed that SF5 treatment led to a higher inhibition in leukemic cell growth, DNA synthesis and in the cell cycle. This pronounced effect compared to HEX must be related to their different chemical composition. HEX contains other compounds that are not present in SF5, which may be contributing to its reduced inhibitory effect by either neutralizing the inhibitory action of SF5 compounds or inducing activation effects.

The inhibition of tumor cell proliferation has been demonstrated for traditional chemotherapeutic agents and potential anti-cancer drugs [22], as observed with MTX in this work. SF5 cytotoxic effects were also demonstrated on other tumor cell lines although they were lower than those on leukemia cells (data not shown). The finding of a terpenic subfraction with higher anti-tumor effect than the hexanic fraction of the original extract is of great value since it could represent a potential anti-tumor agent with fewer side effects. The anti-proliferative effects of SF5 treatment here observed were not due to the presence of ethanol in this subfraction since control cultures treated with ethanol in the same concentration did not exhibit these effects (data not shown).

Cyclin D is the first cyclin expressed by cells in response to favorable growth conditions [1]. In conjunction with CDK4/6, cyclin D mediates the initial phosphorylation of the Retinoblastoma protein (pRb). The kinase activity of the cyclin E/CDK2 complex then acts to maintain pRb in the hyper-phosphorylated state [2]. Any factor affecting these kinase activities could abrogate the normal inactivation of pRb and cause accumulation of cells in the G_1_ phase. The mechanisms involved in the *in vitro* anti-cancer activity of SF5 seem to be similar to those of EEPp [15], leading to the inhibition of cyclin E2 gene expression and resulting in cell cycle arrest in the G1 phase. SF5 treatment also increased cyclin D1 gene expression as did cell exposure to EEPp [15]. Other cell lines have also shown an increase in D1 cyclin expression associated with the inhibition of cell proliferation [23].

SF5 treatment also increased p21gene expression which may be collaborating to cell cycle inhibition. Several reports describe the up-regulation of p21 [24] by anti-cancer drugs. According to the literature, p21 plays a direct role in mediating p53-induced G1 arrest [25]. On the other hand, as K562 cells have a mutation on the *TP53* tumor suppressor gene [26], the effects of increased p21 gene expression on cell cycle arrest in this work must be related to a p53*-*independent mechanism, as seen during cellular senescence [27] and differentiation [26,28]. The increased mRNA expression of both cyclin D1 and p21, observed with SF5 treatment could result in higher levels of these proteins and formation of p21-CDK4-cyclin D1 and/or p21-CDK6-Cyclin D1 complexes, decreasing CDK4 and CDK6-associated kinase activities in K562 cells [29]. Sequestration of cell cycle inhibitors (p21 and p27) is well established for D-cyclins [3]. Furthermore, SF5 exposure inhibited cyclin E mRNA expression that can lead to reduced formation of CDK2-cyclin E protein complex and, consequently, to a reduced CDK2 kinase activity. Cyclin E2 down-regulation has also been reported as an important effect of anti-cancer drugs [30].

The expression of cyclins and cell cycle inhibitors depends on different transcription factors and MAP kinases activities [31-33]. Since ERKs signaling pathway stimulates c-myc expression [5], cyclin E2 gene transcription is activated by c-myc [7], and SF5 treatment inhibited both ERK 1/2 activation and c-myc and cyclin E2 gene expression, the cell cycle arrest observed in this work may be related to the inhibition of MAP kinase phosphorylation and to a reduced level of c-myc.

The up-regulation of cyclin D1 and p21 expression induced by SF5 treatment, despite the reduced activation of ERK 1/2, suggests that the expression of these proteins can be submitted to other regulatory mechanisms. Indeed, p21 and p27 gene expression can be regulated by the transcription factor AP-1 [34] or by promoter methylation [35]. Otherwise, cyclin D1 gene expression is under the regulation of other transcription factors such as AP1 and STATs [36].

In addition, the transcription factor NF-κB, activated in certain cancers [37], is linked to the expression of different genes associated with cell proliferation such as c-myc [38] and E2 [39]. NF-κB has also been an important target for anti-cancer treatment and this study demonstrated that SF5 exposure inhibited NF-κB translocation to the nucleus, probably inhibiting the expression of specific NF-κB targets, as here demonstrated for cyclin E2 and c-myc.

## Conclusions

Concluding, SF5 treatment showed a minor chemical complexity and higher *in vitro* anti-leukemic action than HEX, which could represent a reduction of side effects for a potential anti-tumoral agent. SF5’s anti-leukemic action is linked to the inhibition of intracellular signaling, such as the lower activation of ERK 1/2 and NF-κB and the altered expression of cell cycle regulatory proteins, as demonstrated by increased p21 mRNA levels and down-regulation of c-myc and cyclin E2 expression.

## Competing interest

The authors declare that they have no competing interests.

## Authors’ contributions

PMF and MT carried out the experiments. SKCC designed and coordinated the study. BFC (Cell biology), ARM (Molecular biology), PMC (Cell biology), CMGP (Phytochemistry) and DSR (Flow cytometry) contributed to experiments with their specific expertise. All authors approved the final manuscript.

## Pre-publication history

The pre-publication history for this paper can be accessed here:

http://www.biomedcentral.com/1472-6882/12/231/prepub

## References

[B1] Malumbres M Barbacid M To cycle or not to cycle: a critical decision in cancer Nat Rev Cancer 2001 1 222 231 10.1038/35106065 11902577

[B2] Sarsour EH Kumar MG Chaudhuri L Kalen AL Goswami PC Redox control of the cell cycle in health and disease Antiox Redox Signal 2009 11 2985 3011 10.1089/ars.2009.2513 PMC2783918 19505186

[B3] Lange CA Yee D Killing the second messenger: targeting loss of cell cycle control in endocrine-resistant breast cancer Endoc Relat Cancer 2011 18 C19 C24 10.1530/ERC-11-0112 PMC3924782 21613412

[B4] Anderson DH Role of lipids in the MAPK signaling pathway Prog Lipid Res 2006 45 102 119 10.1016/j.plipres.2005.12.003 16455141

[B5] Verykokakis M Papadaki C Vorgia E Le Gallic L Mavrothalassitis G The RAS-dependent ERF control of cell proliferation and differentiation is mediated by c-Myc repression J Biol Chem 2007 282 30285 30294 10.1074/jbc.M704428200 17699159

[B6] Chang F Steelman LS Shelton JG Lee JT Navolanic PM Blalock WL Franklin R McCubrey JA Regulation of cell cycle progression and apoptosis by the Ras/Raf/MEK/ERK pathway Int J Oncol 2003 22 469 480 12579299

[B7] Beier R Bürgin A Kiermaier A Fero M Karsunky H Saffrich R Möröy T Ansorge W Roberts J Eilers M Induction of cyclin E-cdk2 kinase activity, E2F-dependent transcription and cell growth by Myc are genetically separable events EMBO J 2000 19 5813 5823 10.1093/emboj/19.21.5813 11060032 PMC305784

[B8] Morante M Sandoval J Gómez-Cabrera MC Rodríguez JL Pallardó FV Viña JR Torres L Barber T Vitamin E deficiency induces liver nuclear factor-kappaB DNA-binding activity and changes in related genes Free Radic Res 2005 39 1127 1138 10.1080/10715760500193820 16298738

[B9] Feng B Cheng S Hsia CY King LB Monroe JG Liou HC NF-κB inducible genes BCL-X and cyclin E promote immature B-cell proliferation and survival Cell Immunol 2004 232 9 20 10.1016/j.cellimm.2005.01.006 15922711

[B10] Lavon I Fuchs D Zrihan D Efroni G Zelikovitch B Fellig Y Siegal T Novel mechanism whereby nuclear factor kappaB mediates DNA damage repair through regulation of O(6)-methylguanine-DNA-methyltransferase Cancer Res 2007 67 8952 8959 Erratum in: Cancer Res 2007, 67:10624 10.1158/0008-5472.CAN-06-3820 17875738

[B11] Yazdanparast R Sadeghi H Nucleic acid synthesis in cancerous cells under the effect of gnidilatimonoein from Daphne mucronata Life Sci 2004 74 1869 1876 10.1016/j.lfs.2003.08.039 14761668

[B12] Pio Corrêa M Dicionário das plantas úteis do Brasil e das exóticas cultivadas Ministério da Agricultura. Instituto Brasileiro de Desenvolvimento Florestal. Rio de Janeiro 1984 III 129

[B13] Sabino KCC Castro FA Oliveira JCR Dalmau SRA Coelho MGP Successful treatment of collagen-induced arthritis in mice with hydroalcohol extract of seeds of Pterodon pubescens Phytother Res 1999 13 613 615 10.1002/(SICI)1099-1573(199911)13:7<613::AID-PTR503>3.0.CO;2-D 10548757

[B14] Silva MC Gayer CR Lopes CS Calixto NO Reis PA Passaes CP Paes MC Dalmau SR Sabino KC Todeschini AR Coelho MG Acute and topic anti-edematogenic fractions isolated from the seeds of Pterodon pubescens J Pharm Pharmacol 2004 56 135 141 14980011 10.1211/0022357022485

[B15] Pereira MF Simão TA Dalmau SR Albano RM Coelho MGP Sabino KCC Pterodon pubescens seed extract induces the cell cycle arrest of leukemic cells by deregulating cyclin D1 and E2 mRNA levels Oncol Lett 2010 1 533 536 22966338 10.3892/ol_00000094 PMC3436211

[B16] Fascio M Gilbert B Mors WB Nishida T Two new diterpenes from Pterodon pubescens Benth An Acad Bras Cienc 1970 42 97 101

[B17] Fascio M Mors WB Gilbert B Mahajan JR Monteiro MB Dos Santos Filho D Vichnewski W Diterpenoid furans from Pterodon species Phytochem 1976 15 201 203 10.1016/S0031-9422(00)89084-6

[B18] Vieira CR Marques MF Soares PR Matuda L Oliveira CMA Kato L da Silva CC Guillo LA Antiproliferative activity of Pterodon pubescens Benth. seed oil and its active principle on human melanoma cells Phytomedicine 2008 15 528 532 10.1016/j.phymed.2007.08.003 17913485

[B19] Pereira MF Martino T Dalmau SR Albano RM Pierre-Férézou J Costa SS Coelho MGP Sabino KCC Terpenic subfraction of Pterodon pubescens induces apoptosis of K562 cells by modulating gene expression Oncol Rep 2011 25 215 221 21109979

[B20] Brando-Lima AC Saldanha-Gama RF Pereira CR Villela CG Sampaio AL Monteiro-Moreira AC Henriques MG Moreira RA Barja-Fidalgo C Involvement of phosphatidylinositol-3 kinase-Akt and nuclear factor kappa-B pathways in the effect of frutalin on human lymphocyte Int Immunopharmacol 2005 6 465 472 16428082 10.1016/j.intimp.2005.09.008

[B21] Lowry OH Rosenbrough NJ Farr AL Randall RJ Protein measurement with the Folin phenol reagent J Biol Chem 1951 193 265 275 14907713

[B22] Qurishi Y Hamid A Majeed R Hussain A Qazi AK Ahmed M Zargar MA Singh SK Saxena AK Interaction of natural products with cell survival and signaling pathways in the biochemical elucidation of drug targets in cancer Fut Oncol 2011 7 1007 1021 10.2217/fon.11.69 21823895

[B23] Okabe H Lee SH Phuchareon J Albertson DG McCormick F Tetsu O A critical role for FBXW8 and MAPK in cyclin D1degradation and cancer cell proliferation PLoS One 2006 1 e128 10.1371/journal.pone.0000128 17205132 PMC1762433

[B24] Martin BT Kleiber K Wixler V Raab M Zimmer B Kaufmann M Strebhardt K FHL2 regulates cell cycle-dependent and doxorubicin-induced p21Cip1/Waf1 expression in breast cancer cells Cell Cycle 2007 6 1779 1788 10.4161/cc.6.14.4448 17682292

[B25] Li TM Chen GW Su CC Lin JG Yeh CC Cheng KC Chung JG Ellagic acid induced p53/p21 expression, G1 arrest and apoptosis in human bladder cancer T24 cells Anticancer Res 2005 25 971 979 15868936

[B26] Jiang H Lin J Su ZZ Collart FR Huberman E Fisher PB Induction of differentiation in human promyelocytic HL-60 leukemia cells activates p21 CIP/WAF1/CIP1 expression in the absence of p53 Oncogene 1994 9 3397 3406 7936668

[B27] Noda A Ning Y Venable SF Pereira-Smith OM Smith JR Cloning of senescent cell-derived inhibitors of DNA synthesis using an expression screen Exp Cell Res 1994 211 90 98 10.1006/excr.1994.1063 8125163

[B28] Macleod KF Sherry N Hannon G Beach D Tokino T Kinzler K Vogelstein B Jacks T p53-dependent and independent expression of p21 during cell growth, differentiation, and DNA damage Genes Dev 1995 9 935 944 10.1101/gad.9.8.935 7774811

[B29] Lu MC Yang SH Hwang SL Lu YJ Lin YH Wang SR Wu YC Lin SR Induction of G2/M phase arrest by squamocin in chronic myeloid leukemia (K562) cells Life Sci 2006 78 2378 2383 10.1016/j.lfs.2005.09.048 16310807

[B30] Diaz-Carballo D Malak S Freistühler M Elmaagacli A Bardenheuer W Reusch HP Nemorosone blocks proliferation and induces apoptosis in leukemia cells Int J Clin Pharmacol Ther 2008 46 428 439 18793585 10.5414/cpp46428

[B31] Liu Y Martindale JL Gorospe M Holbrook NJ Regulation of p21 WAF1/CIP1 expression through mitogen-activated protein kinase signaling pathway Cancer Res 1996 56 31 35 8548769

[B32] Lavoie JN L'Allemain G Brunet A Müller R Pouysségur J Cyclin D1 expression is regulated positively by the p42/p44MAPK and negatively by the p38/HOGMAPK pathway J Biol Chem 1996 271 20608 20616 10.1074/jbc.271.34.20608 8702807

[B33] Muise-Helmericks RC Grimes HL Bellacosa A Malstrom SE Tsichlis PN Rosen N Cyclin D expression is controlled post-transcriptionally via a phosphatidylinositol 3-kinase/Akt-dependent pathway J Biol Chem 1998 273 29864 29872 10.1074/jbc.273.45.29864 9792703

[B34] Barboule N Lafon C Chadebech P Vidal S Valette A Involvement of p21 in the PKC-induced regulation of the G2/M cell cycle transition FEBS Lett 1999 444 32 37 10.1016/S0014-5793(99)00022-8 10037143

[B35] Furukawa Y Sutheesophon K Wada T Nishimura M Saito Y Ishii H Furukawa Y Methylation silencing of the Apaf-1 gene in acute leukemia Mol Cancer Res 2005 3 325 334 10.1158/1541-7786.MCR-04-0105 15972851

[B36] Joyce D Albanese C Steer J Fu M Bouzahzah B Pestell RG NF-κB and cell cycle regulation: the cyclin connection Cytok and Growth Factor Reviews 2001 12 73 90 10.1016/S1359-6101(00)00018-6 11312120

[B37] Karin M Cao Y Greten FR Li ZW NF-kappaB in cancer: from innocent bystander to major culprit Nat Rev Cancer 2002 2 301 310 10.1038/nrc780 12001991

[B38] Chow JM Liu CR Lin CP Lee CN Cheng YC Lin SH Liu HE Down-regulation of c-Myc determines sensitivity to 2-methoxyestradiol–induced apoptosis in human acute myeloid leukemia Exper Hematol 2008 36 140 148 10.1016/j.exphem.2007.10.004 18206725

[B39] Hsia CY Cheng S Owyang AM Dowdy SF Liou HC c-Rel regulation of the cell cycle in primary mouse B lymphocytes Int Immunol 2002 14 905 916 10.1093/intimm/dxf055 12147627

